# COVID-19 and mental health among older people in Sweden

**DOI:** 10.1017/S104161022000143X

**Published:** 2020-07-08

**Authors:** Ingmar Skoog

**Affiliations:** 1 Section of Psychiatry and Neurochemistry, Institute of Neuroscience and Physiology, Sahlgrenska Academy, University of Gothenburg, Gothenburg, Sweden; 2 Region Västra Götaland, Sahlgrenska University Hospital, Psychiatry, Cognition and Old Age Psychiatry Clinic, Mölndal, Sweden

In Sweden, the pandemic started to influence the country in mid-March. Sweden has not had a complete lockdown, but a more relaxed restriction, where people are allowed to go outside their homes, are able to go to work but recommended to work from home, and where restaurants and shops remained open. Schools for the three youngest years have remained open since the beginning of the pandemic, and in June all schools, including universities, were allowed to reopen. In practice, people have followed the restrictions and been very reluctant to go to restaurants and shops, and most people work from home. Recommendations of distance and frequent hand washing have applied.

However, more strict restrictions apply for those above age 70. The Public Health Agency announced, in a very patronizing way, on March 16, that the rest of the country should protect the weak and frail ‘oldest-old’, defined as all those aged 70 years and above. It was strongly advised that children and grandchildren should avoid visiting their parents and grandparents, and only keep contact by phone or Internet. This started a wave of ageism going through the country. Older people were verbally abused for walking outdoors or going to shops, and thus jeopardizing the Swedish system of infection control.

Regarding mental health care for older people, all non-acute physical visits to mental health care facilities were abandoned for people aged 70 and above. Contacts are instead performed by telephone or Internet. In cases where a physical visit is regarded as necessary, a home visit is the second choice, but this was only applied in severe cases. The third choice is a visit to the outpatient department. Before a visit to the outpatient department, all patients are asked whether they have any signs of infection. All waiting rooms are organized to have distance between patients. Inpatient care has been avoided as much as possible. The mental health consequences of these changes are still not clear. There has been no increase observed in visits to the outpatient department of geriatric psychiatry during the pandemic. Initially, there was a clear reduction of visits to all emergency departments, including geriatric psychiatry. There is also less patients at the inpatient departments of geriatric psychiatry (a decline with one-fourth). Now when the strong restrictions regarding people above age 70 seem to continue for a long period of time, a more liberal attitude to home visits and visits to the outpatient departments have developed, as many older people with mental disorders suffer much due to the long-term quarantine. At these visits, the staff keep distance and strong hygiene is applied.

In March 21, several leading psychogeriatricians and geriatricians wrote a debate article in one of Sweden’s leading national newspapers pointing to the risk of spreading of the coronavirus into old age care facilities and home care (Skoog *et al.*, 2020). The authors pointed out that there is a large disparity in old age care in Sweden, as it is organized in 290 communities, and among these in a mix of private and public organizations. The debate article therefore suggested that a national coordinator regarding coronavirus disease 2019 (COVID-19) in older people should be appointed. In addition, those in old age homes are often very frail and sick, and 70–90% have dementia. The same applies to people who receive home care, where two-thirds may have dementia. The staff are often not well educated and have uncertain employment status, often on a per-hour basis. There is often a large circulation of staff, where an old person in home care may have 16 different carers during a 2-week period. In addition, each carer may have to take care of 15–20 older persons per working day. It is also obvious that the interaction between staff and care recipient is very close, as many service users need help with clothing, hygiene, toileting, etc. In March came a report that only 60% of old age care facilities followed basic recommendations regarding hygiene (Socialstyrelsen, 2020a). There was also an initial lack of personal protective equipment, and no availability of COVID-19 tests during the first 10 weeks. Add to this that old age care is organized under social work, not under health care. This might be one reason, why there still are reports that staff move between patients with COVID-19 infection and older residents without infection. Despite a number of debate articles and attempts to contact authorities, neither officials nor media took any serious notice of the possible problems with old age care, except that the government came with a law that prohibited all visits from relatives to residents living at old age care facilities. In mid-April came reports that 50% of deaths from COVID-19 came from people living at old age homes and another 25% from people who had home care (Socialstyrelsen, 2020b). After that, media interest in old age care exploded, and it was announced as the major failure of the Swedish COVID-19 strategy.

After a couple of weeks, some debate also started regarding the mental health consequences of isolating old people in their homes and of abandoning visits from relatives to people living in old age care facilities. It should be noted that already before the pandemic, people living in old age care facilities reported more loneliness and had a higher prevalence of depression than people living at home. So far, there is no real discussion on whether the mental health consequences of long-term isolation outweigh the risk of getting infected with the coronavirus.

It has been reported that many persons above age 70 now are getting depressed and call centers, where people call if they have suicidal feelings, are receiving an increasing number of calls from older people (Bjarnefors and Pettersson, 2020). The general increase in calls is reported to be 60%, and a large proportion of the increase is accounted for by seniors. The reason for the increase in mental health problems among seniors is probably not only the isolation itself, but also the increasing ageism observed in Sweden, as noted above. Ageism was a problem in Sweden already before the pandemic, where only a few members of the parliament are above age 65, and where people already in their 40s are thought to be too old to get a new employment. Before the pandemic, a more positive opinion about older people had slowly emerged, where the fact that seniors today are much more physically and mentally healthy, have better cognitive and physical function, and are more active than a couple of decades ago, led to the slogan “70 is the new 50.” There was also a serious discussion on whether this should lead to an increase in the compulsory retirement age in Sweden. It was a chock for many active seniors to suddenly be regarded as weak and helpless. However, the isolation itself also has obviously negative consequences, especially when the rest of the population are allowed to continue a normal, albeit somewhat restricted, life. There were a lot of private and public initiatives to ease the isolation for older people, such as help with shopping and organization of walking tours for older people. However, this would not ease the stigma for older persons used to be independent and used to live an active and responsible life.

In June, it was expected that the Public Health Agency would ease the restrictions for healthy persons above age 70. Instead, to the disappointment of many seniors, it was decided that the restrictions should continue. At the same time, less restrictions were applied for people below age 70. However, the Public Health Agency has given more details regarding social isolation, and given older people the ability to meet outdoors with friends and family if appropriate distance is applied. Recently, the recommendation against traveling within Sweden was taken away, and this is now permitted for people of all ages, including also those above age 70. This gives at least some relief, as seniors now are able to travel to their summerhouses, a very common activity in Sweden.

The ban against visitors to old age care facilities will as it looks now continue over the summer. There are already reports of mental health consequences, both for relatives and residents. The possibility to meet outdoors applies also to people living at old age homes, but many are not able to do this. As it seems now, most of the spread of the coronavirus to old age homes has come via staff. It could be discussed how much the risk would increase if individual symptom-free relatives were allowed to visit under strict rules regarding distance and hygiene.

In summary, ageism in Sweden, and the special rules applied to otherwise healthy seniors leading to stigma for older people, may increase the risk of mental health problems among previously healthy older people. This is reflected by the increasing number of calls to call centers for people with suicidal feelings. The risk for mental health consequences also applies to older people living at old age care facilities who are isolated from relatives for months. The mental health consequences of long-term restrictions applied to people above age 70 has not been seriously discussed in Sweden despite that these restrictions seem to continue over the summer.

In relation to ongoing or proposed strategies or solutions as part of the pandemic response to address mental health of older adults in Sweden, there are currently no consistent public strategies. Most efforts are done by pensioner and volunteer organizations and charities, in some instances by public support. In relation to ageism, there was a debate about this already before the pandemic. The parliament, which includes only six members of parliament above age 65 out of a total of 349, appointed a committee to address this question a couple of years ago. In relation to the pandemic, there is no real discussion or willingness from authorities to address the increasing ageism observed since the start of the pandemic, and its consequences for mental health in old age.
